# Challenges faced by the HIV testing system in low- and middle-income countries

**DOI:** 10.4102/ajlm.v12i1.1974

**Published:** 2023-01-31

**Authors:** Rachel S. Kamgaing, Yagai Bouba, Samuel M. Sosso, Jeremiah E. Gabisa, Aubin Nanfack, Joseph Fokam, Laure Ngono, Nadine Fainguem, Michel C.T. Tommo, Krystel N. Zam, Junie F. Yimga, Désiré K. Takou, Alexis Ndjolo

**Affiliations:** 1 Medical Analysis Laboratory, Chantal Biya International Reference Centre for Research on HIV/AIDS Prevention and Management, Yaoundé, Cameroon; 2 Section Appui au Secteur Sante, National AIDS Control Committee, Yaoundé, Cameroon; 3 Chantal Biya International Reference Centre, Yaoundé, Cameroon; 4 Department of Experimental Medicine, Faculty of Medicine, University of Rome Tor Verga, Rome, Italy; 5 Medical Laboratory Analysis, Chantal Biya International Reference Centre for Research on HIV/AIDS Prevention and Management, Yaoundé, Cameroon; 6 Laboratory of Immunology and Microbiology, Chantal Biya International Reference Centre for Research on HIV/AIDS Prevention and Management, Yaoundé, Cameroon; 7 Laboratory of Virology, Chantal Biya International Reference Centre for Research on HIV/AIDS Prevention and Management, Yaoundé, Cameroon; 8 Department of Medical Laboratory Sciences, Faculty of Health Sciences, University of Buea, Buea, Cameroon; 9 Laboratory of Virology, Centre Pasteur du Cameroun, Yaoundé, Cameroon; 10 Catholic University of Central Africa, Yaoundé, Cameroon; 11 Centre de Biotechnologie de l’Université de Yaoundé 1, Yaoundé, Cameroon; 12 Chantal Biya International Reference Centre for Research on HIV/AIDS Prevention and Management, Yaoundé, Cameroon

**Keywords:** HIV diagnosis, counselling, HIV testing system, testing technologies, algorithm, Cameroon

## Abstract

**Introduction:**

Determining the HIV status of some individuals remains challenging due to multidimensional factors such as flaws in diagnostic systems, technological challenges, and viral diversity. This report pinpoints challenges faced by the HIV testing system in Cameroon.

**Case presentation:**

A 53-year-old male received a positive HIV result by a rapid testing algorithm in July 2016. Not convinced of his HIV status, he requested additional tests. In February 2017, he received a positive result using ImmunoComb^®^ II HIV 1 & 2 BiSpot and Roche cobas electrochemiluminescence assays. A sample sent to France in April 2017 was positive on the Bio-Rad GenScreen™ HIV 1/2, but serotyping was indeterminate, and viral load was < 20 copies/mL. The Roche electrochemiluminescence immunoassay and INNO-LIA HIV I/II Score were negative for samples collected in 2018. A sample collected in July 2019 and tested with VIDAS^®^ HIV Duo Ultra enzyme-linked fluorescent assay and Geenius™ HIV 1/2 Confirmatory Assay was positive, but negative with Western blot; CD4 count was 1380 cells/mm^3^ and HIV proviral DNA tested in France was ‘target-not-detected’. Some rapid tests were still positive in 2020 and 2021. Serotyping remained indeterminate, and viral load was ‘target-not-detected’. There were no self-reported exposure to HIV risk factors, and his wife was HIV-seronegative.

**Management and outcome:**

Given that the patient remained asymptomatic with no evidence of viral replication, no antiretroviral therapy was initiated.

**Conclusion:**

This case highlights the struggles faced by some individuals in confirming their HIV status and the need to update existing technologies and develop an algorithm for managing exceptional cases.

## Background

HIV-1 infection is still a significant public health concern in sub-Saharan Africa, where over two-thirds of the world’s burden is found. Nevertheless, the number of new infections has dropped from about 1 950 000 in 2000 to approximately 870 000 in 2020.^1^ Following this remarkable achievement, the Joint United Nations Programme on HIV/AIDS 2025 goals of 95-95-95 and eradicating HIV/AIDS by 2030 are now feasible targets.^2^ Efforts in early diagnosis and the scale-up of antiretroviral therapy use have played a prominent role in this achievement. However, several issues remain to be addressed,^3^ including the high diversity of circulating HIV strains and the limited availability of appropriate and advanced technology to determine HIV status of exceptional cases.^4^ For example, in Cameroon, the HIV testing algorithm comprises Determine HIV 1/2 (Abbott Molecular Inc, Des Plaines, Illinois, United Sates), Hexagon HIV 1/2 (Human Biochemica, Wiesbaden, Germany), and OraQuick HIV 1/2 (OraSure Technologies Inc, Petchabun, Thailand). However, the weakness of the sample referral system, and the limited local availability of advanced technologies, among other reasons, continue to be a challenge in confirming the HIV status of some individuals in several African countries, including Cameroon.^5^

Furthermore, poor adherence to the national guidelines of an HIV testing programme also significantly affects the consistency and reliability of HIV results.^6^ This poor adherence might play a role in some individuals’ denial of HIV results.^7,8^ Thus, we present this case report to outline one of the challenges some individuals face in confirming their HIV status in Cameroon.

## Ethical considerations

This diagnostic report was conducted per the Declaration of Helsinki principles and national regulations. The patient provided a signed informed consent, declaring his will to freely participate and authorising the usage of his medical laboratory findings in this report. No patient-identifying information was used throughout the paper.

## Case presentation

**FIGURE 1:**
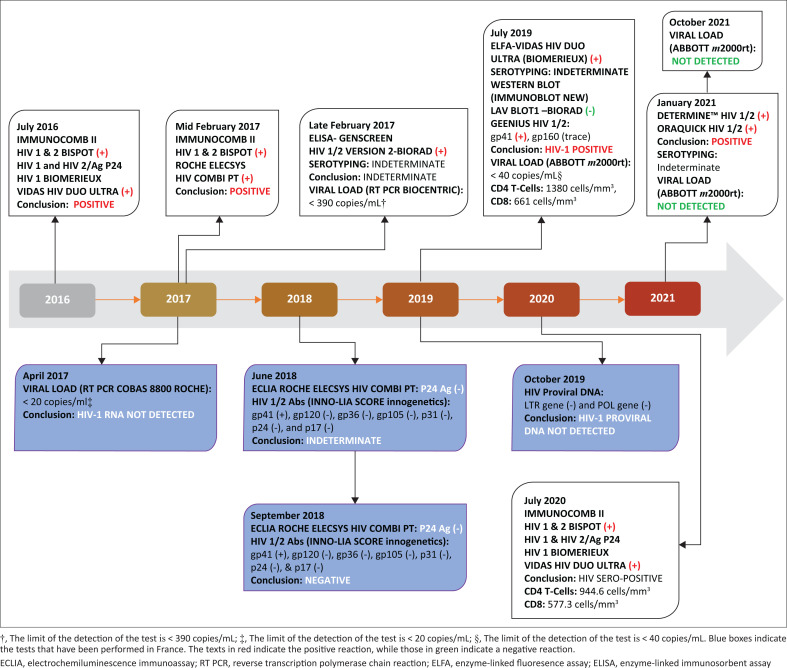
Summary of the testing performed in the patient’s history from 2016 to 2021, Cameroon.

We present the case of a 53-year-old married and asymptomatic man who received a confirmatory positive HIV diagnosis in 2016. The different tests performed, the methods used, and the corresponding time point are summarised in Figure 1.

After a consultation, he was determined to be HIV-positive following an HIV test request using rapid diagnostic tests: Determine HIV 1/2 (Abbott Molecular Inc, Des Plaines, Illinois, United Sates), Hexagon HIV 1/2 (Human Biochemica, Wiesbaden, Germany), and OraQuick (OraSure Technologies, Inc, Petchabun, Thailand); included in the Cameroon screening algorithm as per the World Health Organization HIV testing guidelines. However, his wife tested HIV-seronegative. Not convinced of his status, he sought another test in a laboratory with a more advanced technique. The laboratory tested his sample collected in July 2016; the results were positive using the ImmunoComb^®^ II HIV 1 & 2 BiSpot (Orgenics Ltd, Yavne, Israel) and VIDAS^®^ HIV Duo Ultra (bioMérieux, Marcy-l’Etoile, France) tests. A few months later, in another laboratory, an HIV-positive status was obtained on a sample collected in February 2017 using the ImmunoComb^®^ II HIV 1 & 2 BiSpot (Orgenics Ltd, Yavne, Israel) and the Elecsys^®^ HIV combi PT electrochemiluminescence immunoassay techniques (Roche Diagnostics GmbH, Mannheim, Germany) (Figure 1).

About two weeks later, he presented at one of Cameroon’s national reference laboratories in Yaoundé for serotyping and viral load testing. Unfortunately, the serotyping test was indeterminate, and the viral load test was < 390 copies/mL by reverse transcription polymerase chain reaction (BioCentric Inc, Collingswood, New Jersey, United States), while the enzyme-linked immunosorbent assay, Genscreen HIV1/2 (Bio-Rad Diagnostics Ltd, Hercules, California, United States), was positive on this same sample. About six weeks later, the viral load test was repeated in France with a more sensitive reverse transcription polymerase chain reaction cobas^®^ 8800 assay (Roche Diagnostics Ltd, New South Wales, Australia), and the result was < 20 copies/mL (which corresponds to the lower limit of the detection of this test).

Following these results, new samples were ordered by the consultant and sent to France again; the Elecsys^®^ HIV combi PT electrochemiluminescence immunoassay and INNO-LIA HIV 1/2 Score assay (Fujirebio Diagnostics Inc, Malvem, Pennsylvania, United States) were performed on two samples collected over a three-month interval; the first gave an indeterminate result and the second was concluded as a negative result.

This result comforted the patient in his doubt about his HIV status, but on a new sample collected in July 2019, the VIDAS^®^ HIV Duo Ultra enzyme-linked fluorescent assay (bioMérieux, France), and Geenius™ HIV1/2 assay (Geenius™ Diagnostic, Inc, Redmond, Washington, United States) were still positive. His viral load was < 40 copies/mL, the Western blot was negative, and serotyping was indeterminate. The patient remained asymptomatic with a normal immunological status (CD4 T-Cells: 1380 cells/mm^3^, CD8 T-cells: 661 cells/mm^3^). HIV proviral DNA was not detectable in a sample collected on 02 October 2019. About 10 months later, serological tests were still HIV-positive, with a CD4 count of 945 cells/mm^3^.

In January 2021, he was sent to the Chantal Biya International Research Centre for the Prevention and Management of HIV/AIDs, Yaoundé, for viral load testing using the Abbott *m*2000rt RealTime HIV-1 assay (Abbott Molecular Inc, Des Plaines, Illinois, United Sates), and the result was ‘target-not-detected’. Of note, on this same sample, Determine and OraQuick were positive, while serotyping remained indeterminate. As of October 2021, his viral load remained ‘target-not-detected’, and he is still asymptomatic. Noteworthy, he stated that he did not have any chronic diseases, nor had he engaged in any HIV-related risk behaviour. Furthermore, his serological tests were negative for hepatitis B surface antigen (HBsAg) and hepatitis C virus antibodies using rapid tests.

## Management and outcomes

So far, given that the patient remained asymptomatic with no evidence of viral replication, no antiretroviral therapy was initiated. We collected a new sample in our laboratory on 08 October 2021, and performed rapid testing according to the national algorithm, serotyping and HIV-1 viral load, but the outcome was serology-positive, viral load ‘target-not-detected’. However, he was advised to avoid any HIV risk behaviour, and we continue to monitor his serological and virological status.

## Discussion

Efforts have been made towards improving laboratory medicine in Africa, especially after the 2008 Maputo declarations. Some authors think a remarkable transformation has occurred in some areas of laboratory medicine, especially in those targeting the fight against HIV/AIDS in sub-Saharan Africa.^3,9^ Nonetheless, numerous challenges, including weaknesses in the quality management systems, laboratory networks, human resources, and technological issues, must be appropriately addressed.^9,10,11,12^ Despite testing progress, many factors influence the acceptance or refusal of provider-initiated testing and including self-trust, not being at risk for HIV, not being ready, and insufficient privacy and counselling.^8^ The aforementioned factors could have also influenced the man’s denial of his HIV test results of 08 July 2016 and 15 February 2017. Moreover, a study conducted in Cameroon between 2013 and 2014 reported that using lay counsellors in HIV testing units is a setback to delivering quality HIV testing services, where counselling and acceptance are cornerstones.^7^

Confirming HIV status is still challenging in some cases, especially in Western African countries, characterised by many HIV-1 subtypes, as highlighted by Aghokeng et al., who found an inaccurate diagnosis of specific HIV-1 subtypes, notably in Group M and O.^5^ Therefore, there is a need for more sophisticated confirmatory tests.^5^ In the case described here, most of these tests, such as the sensitive viral load threshold test and the detection of HIV-1 proviral DNA, were performed in Europe. Complex cases are sometimes encountered in diagnostic laboratories, but unfortunately, due to the absence or limited access to these tests, patients are without any conclusive HIV status.

Therefore, more sensitive diagnostics are essential, especially in this ‘test and treat’ era, where all patients testing seropositive are encouraged to start antiretroviral treatment as soon as possible to limit false positive cases. False positive tests have been reported in some developing countries, including Cameroon.^13,14^ A well-functioning system to detect complex cases that might require further confirmation should be implemented to avoid unnecessary treatment.

We advocate that national public health laboratories should be equipped and strengthened to handle complex situations. Furthermore, besides the financial cost, there is the psycho-social burden posed on the patient due to an uncertain HIV status. The psycho-social burden goes beyond the individual level and affects the family and socio-economic dimensions.

There are still unresolved issues about the HIV status of this man. The antibodies are persistently reactive, serotyping is continuously indeterminate, and the viral load measurement is ‘target-not-detected’. At this stage, the best way to demonstrate the presence of the virus would be to isolate it, but this will be challenging because of the undetectable viral load. It is still to be demonstrated, but we might also hypothesise that this man might be a rare case of an ‘elite controller’ or a ‘long-term non-progressor’.

### Conclusion

Overall, this case highlights the struggles faced by some individuals in confirming their HIV status. In addition, it calls for the need to adapt existing technologies and develop an algorithm for managing special cases in low- and middle-income countries.

## References

[1] UNAIDS_Fact Sheet [homepage on the Internet]. [cited 2021 Oct 16]. Available from: https://www.unaids.org/sites/default/files/media_asset/UNAIDS_FactSheet_en.pdf

[2] UNAIDS issues new fast-track strategy to end AIDS by 2030 – EGPAF [homepage on the Internet]. Elizabeth Glaser Pediatric AIDS Foundation; 2014 [cited 2021 Mar 26]. Available from: https://www.pedaids.org/2014/11/20/unaids-issues-new-fast-track-strategy-to-end-aids-by-2030/

[3] Nkengasong JN, Yao K, Onyebujoh P. Laboratory medicine in low-income and middle-income countries: Progress and challenges. Lancet. 2018;391(10133):1873–1875. 10.1016/S0140-6736(18)30308-8 29550031 PMC5962422

[4] Ragupathy V, Zhao J, Wood O, et al. Identification of new, emerging HIV-1 unique recombinant forms and drug-resistant viruses circulating in Cameroon. Virol J. 2011;8:185. 10.1186/1743-422X-8-185 21513545 PMC3118203

[5] Aghokeng AF, Mpoudi-Ngole E, Dimodi H, et al. Inaccurate diagnosis of HIV-1 group M and O is a key challenge for ongoing universal access to antiretroviral treatment and HIV prevention in Cameroon. PLoS One. 2009;4(11):e7702. 10.1371/journal.pone.0007702 19893738 PMC2768789

[6] Consolidated guidelines on HIV testing services: 5Cs: Consent, confidentiality, counselling, correct results and connection 2015 [homepage on the Internet]. World Health Organization; 2015 [cited 2021 Mar 26]. Available from: https://pubmed.ncbi.nlm.nih.gov/26378328/26378328

[7] Ngangue P, Gagnon M-P, Bedard E. Challenges in the delivery of public HIV testing and counselling (HTC) in Douala, Cameroon: Providers perspectives and implications on quality of HTC services. BMC Int Health Hum Rights. 2017;17(1):9. 10.1186/s12914-017-0118-2 28390398 PMC5385024

[8] Abdurahman S, Seyoum B, Oljira L, Weldegebreal F. Factors affecting acceptance of provider-initiated HIV testing and counselling services among outpatient clients in selected health facilities in Harar town, Eastern Ethiopia. HIV AIDS (Auckl). 2015;7:157–165. 10.2147/HIV.S81649 26028979 PMC4440576

[9] Nkengasong JN, Mbopi-Keou F-X, Peeling RW, et al. Laboratory medicine in Africa since 2008: Then, now, and the future. Lancet Infect Dis. 2018;18(11):e362–e367. 10.1016/S1473-3099(18)30120-8 29980383 PMC13081755

[10] World Health Organization. The Maputo declaration on strengthening of laboratory systems [homepage on the Internet]. [cited 2021 Oct 16]. Available from: https://www.who.int/publications/m/item/the-maputo-declaration-on-strengthening-of-laboratory-systems

[11] Williams J, Umaru F, Edgil D, Kuritsky J. Progress in harmonizing tiered HIV laboratory systems: Challenges and opportunities in 8 African countries. Glob Health Sci Pract. 2016;4(3):467–480. 10.9745/GHSP-D-16-00004 27688718 PMC5042701

[12] Ondoa P, Van Der Broek A, Jansen C, De Bruijn H, Schultsz C. National laboratory policies and plans in sub-Saharan African countries: Gaps and opportunities. Afr J Lab Med. 2017;6(1):578. 10.4102/ajlm.v6i1.578 28879152 PMC5566126

[13] Kosack CS, Shanks L, Beelaert G, et al. HIV misdiagnosis in sub-Saharan Africa: Performance of diagnostic algorithms at six testing sites. J Int AIDS Soc. 2017 Jul 3;20(1):21419. 10.7448/IAS.20.1.21419 28691437 PMC5515032

[14] Coleman SM, Gnatienko N, Lloyd-Travaglini CA, et al. False-positive HIV diagnoses: Lessons from Ugandan and Russian research cohorts. HIV Clin Trials. 2018 Feb;19(1):15–22. 10.1080/15284336.2018.1429846 29384717 PMC5949866

